# The conservative treatment of congenital scoliosis with hemivertebra: report of three cases

**DOI:** 10.1186/1748-7161-8-S1-O51

**Published:** 2013-06-03

**Authors:** AG Aulisa, V Guzzanti, C Perisano, G Scudieri, L Bocchino, S Teramo, L Aulisa

**Affiliations:** 1 Orthopaedic Department, Children's Hospital Bambino Gesù, Rome, Italy; 2 Department of Orthopaedics, University Hospital "Agostino Gemelli", Catholic University of the Sacred Heart, Rome, Italy

## Background

Scoliosis is the most common congenital disorder. The vertebral disorder can be due to failure of formation, segmentation or their combination. Complete formation failure results in hemivertebra that can cause asymmetrical growth and deformity. The etiopathogenesis of congenital scoliosis is still unclear. Twenty-five percent of congenital curves do not progress, 25% undergoes mild progression, while the remaining 50% evolve rapidly and require treatment. Hemivertebra, in the thoraco-lumbar area, display faster rates of progression than those in the lumbar region. The treatment can be either conservative, or surgical. Usually, in rigid and short curves bracing is not recommended, whereas it can be useful for the treatment of secondary curves.

## Aim

To evaluate the efficacy of bracing in congenital scoliosis with hemivertebra.

## Methods

From our database, we identified three patients with congenital scoliosis with hemivertebra. One was 10-year-old at the time of diagnosis and had a hemivertebra localized in L4 with a thoraco-lumbar curve T11-L3. The other one was 6-years-old at the time of diagnosis and had a hemivertebra localized in L2, with a thoraco-lumbar curve T11-L4. The last one was 4-years-old at the time of diagnosis and had a hemivertebra localized in L2, with a thoraco-lumbar curve T11-L3.

## Results

The first patient was treated with a Milwaukee brace, the second with a Boston brace and the third with a PASB brace. The Cobb angles at the beginning were 23°, 53° and 25°, respectively. At the end of treatment, the Cobb angles were18°, 33° and 11°, respectively. At 2 years of follow-up, the curves were 20°, 35° and 13° degrees, respectively.

## Conclusions

Conservative treatment can be considered a valid means to treat not only the patients with congenital scoliosis with hemivertebra who refuse surgery.
